# Dose-dependent antiviral effects of glycyrrhizin, curcumin, and harmaline against clinical SARS-CoV-2 isolates, including D614G, Omicron BA.5, and Omicron XBB.1

**DOI:** 10.1186/s12906-026-05253-1

**Published:** 2026-01-22

**Authors:** Rabea Grüneberg, Isabel Zydek, Carina Elsner, Evelyn Scheiermann, Ulf Dittmer, Folker Meyer, Ivana Kraiselburd, Hana Rohn, Oliver Witzke, Laura Thümmler, Adalbert Krawczyk

**Affiliations:** 1 Department of Infectious Diseases, West German Centre of Infectious Diseases, University Medicine Essen, University Hospital Essen, University Duisburg-Essen, Essen, 45147 Germany; 2 Institute for Virology, University Hospital Essen, University Duisburg-Essen, Essen, 45147 Germany; 3 Institute for Artificial Intelligence in Medicine, University Duisburg-Essen, Essen, 45147 Germany

**Keywords:** Glycyrrhizin, Curcumin, Harmaline, SARS-CoV-2, COVID-19

## Abstract

**Background:**

SARS-CoV-2 remains a major global health challenge, as infection can lead to potential life-threatening conditions such as COVID-19. Emerging variants of the virus are characterized by higher transmission rates and immune escape mutations, enabling them to evade vaccine-induced immunity. Existing treatment options, including monoclonal antibodies, are often variant-specific and not widely accessible, especially in low- and middle-income countries. Natural compounds derived from medicinal herbs and green tea have demonstrated antiviral activity against various viruses and may offer promising, variant-independent therapeutic potential.

**Methods:**

In this study, we examined the antiviral activity of four plant-derived compounds: glycyrrhizin, curcumin, harmaline, and (-)-epigallocatechin. The compounds were tested in vitro against SARS-CoV-2 D614G, Omicron BA.5, and Omicron XBB.1. The antiviral efficacy was assessed at subtoxic concentrations to evaluate potential therapeutic applicability.

**Results:**

All tested compounds showed effective neutralization of SARS-CoV-2 D614G, Omicron BA.5, and Omicron XBB.1 at subtoxic concentrations. In particular, glycyrrhizin, curcumin, and harmaline exhibited potent antiviral activity across all tested variants.

**Conclusions:**

Our findings support the potential of glycyrrhizin, curcumin, and harmaline as variant-independent treatment candidates for COVID-19. However, further clinical studies are necessary to validate their efficacy and safety in vivo.

## Background

 Infection with severe acute respiratory syndrome coronavirus 2 (SARS-CoV-2) continues to cause life-threatening conditions, including coronavirus disease 2019 (COVID-19) and acute respiratory distress syndrome (ARDS), particularly in low- and middle-income countries [1, 2]. Furthermore, long COVID remains a largely underestimated global public health challenge [3, 4]. The rapid approval of several vaccines—including those targeting Omicron variants—and the lower lethality of Omicron compared to previous variants have contributed to a significant reduction in hospitalization rates [5, 6]. However, public opposition to vaccination is increasing, and instances of vaccine fatigue are becoming more widespread [7, 8]. In addition, the emergence of new SARS-CoV-2 variants has led to an increase in breakthrough infections [9, 10]. Current medical treatments include monoclonal antibodies, which are largely variant-specific, require intravenous or subcutaneous administration, and have shown reduced efficacy against emerging SARS-CoV-2 variants such as Omicron BA.5 and XBB.1 [11–13]. Moreover, antiviral agents such as molnupiravir and Paxlovid (nirmatrelvir/ritonavir) remain expensive, have limited global accessibility, and require ongoing adaptation to evolving SARS-CoV-2 variants [14].The development of novel, variant-independent, and widely accessible antivirals for SARS-CoV-2 remains urgently needed.

Bioactive compounds from medicinal herbs and green tea have been extensively studied for their antiviral and antimicrobial properties. These natural agents may thus provide a cost-effective, variant-independent therapeutic strategy, particularly in low- and middle-income countries, where access to novel pharmaceutical therapies remains restricted [15–17]. Glycyrrhizin, curcumin, harmaline, and (-)-epigallocatechin (EGC) exhibited broad-spectrum antimicrobial and antiviral activities against distinct pathogens. Glycyrrhizin, a compound found in licorice root, has demonstrated antiviral effects against herpes simplex virus (HSV) and other viruses [18, 19]. Curcumin, the primary active component of turmeric, has shown inhibitory effects on the replication of a wide range of viruses, including human immunodeficiency virus (HIV), Zika virus, enterovirus 71, human respiratory syncytial virus (RSV), and HSV-2 [20–23]. Harmaline, an alkaloid, is reported to possess antiviral activity against human influenza viruses, HSV-1 and HSV-2 [24, 25]. EGC, a major catechin in green tea, exerted antibacterial, antioxidant, and antiviral effects, and has been shown to inhibit the replication of several viruses including HIV [26]. Some of the aforementioned compounds have demonstrated antiviral activity against seasonal coronaviruses and early variants of SARS-CoV-2 [27–30]. However, it remains unclear whether these agents are also effective against newer SARS-CoV-2 variants and could therefore be considered as adjunctive, variant-independent treatments. Therefore, we investigated the antiviral activity of glycyrrhizin, curcumin, harmaline, and (-)-epigallocatechin against the SARS-CoV-2 variants D614G, Omicron BA.5, and XBB.1.

## Methods

### Cells and viruses

A549-AT cells, a human lung carcinoma cell line (ATCC CCL-185) stably transfected with the receptors angiotensin converting enzyme 2 (ACE2) and transmembrane protease serine subtype 2 (TMPRSS2), were kindly provided by Sandra Ciesek and Marek Widera (Institute of Virology, Frankfurt, Germany) and cultured in Minimum Essential Medium (MEM; Thermo Fisher Scientific, Waltham, MA, USA) supplemented with 10% fetal bovine serum (FBS; Biowest, Nuaillé, France), 100 U/mL penicillin, and 0.1 mg/mL streptomycin (both from Thermo Fisher Scientific) [31]. The cells were cultivated at 37 °C with 5% CO₂ in a standard cell culture incubator. The increased expression of both receptors ensures stable infection of the cells with different SARS-CoV-2 variants, allowing the examination of the agents’ antiviral efficacy against various SARS-CoV-2 variants [31]. The clinical SARS-CoV-2 isolates D614G and its Omicron variants BA.5 and XBB.1 were derived from nasopharyngeal swabs of COVID-19 patients hospitalized at the University Hospital Essen, as described previously [9, 32, 33]. The virus isolation has been approved by the ethics committee of the medical faculty of the University of Duisburg-Essen (approval no. 20–9374-BO). The SARS-CoV-2 variants were identified by sequencing the spike gene and comparing them with the WHO list of variants of concern [34]. The viruses were grown on A549-AT cells and stored at −80 °C. Viral titers were determined using an endpoint dilution assay and calculated as the 50% tissue culture infective dose (TCID₅₀)/mL, as previously described [33].

### Antiviral agents

All tested active ingredients were dissolved, diluted, and stored at − 20 °C. Curcumin (diferuloylmethane; Sigma-Aldrich, Darmstadt, Germany) was prepared by dissolving 10 mg in 0.5 mL dimethyl sulfoxide (DMSO; Carl Roth, Karlsruhe, Germany), followed by a 1:10 dilution in Minimum Essential Medium (MEM) supplemented with 2% fetal bovine serum (FBS), 100 U/mL penicillin, and 0.1 mg/mL streptomycin (Thermo Fisher Scientific). Glycyrrhizin (glycyrrhizin ammonium salt; Sigma-Aldrich, Darmstadt, Germany) was dissolved at a concentration of 400 mg in 4 mL of 100% ethanol (Sigma-Aldrich, Darmstadt, Germany), and the pH was adjusted to 7 using 1 mol/L sodium hydroxide (NaOH; Sigma-Aldrich, Darmstadt, Germany). The solution was then diluted 1:10 in MEM supplemented as described above. (−)-Epigallocatechin (from green tea; Sigma-Aldrich, Darmstadt, Germany) was dissolved by adding 5 mg to 400 µL of DMSO (Carl Roth), then diluted 1:10 in supplemented MEM. Harmaline (Sigma-Aldrich, Darmstadt, Germany) was prepared by dissolving 5 mg in 1 mL of DMSO (Carl Roth), followed by a 1:10 dilution in supplemented MEM.

### Neutralization assay

The antiviral activities of curcumin, glycyrrhizin, (-)-epigallocatechin (EGC), and harmaline were evaluated using a cell culture-based neutralization assay, followed by an endpoint dilution assay to determine their inhibitory effects on viral replication. Therefore, A549-AT cells were seeded in a 24-well plate (at 1.8 × 10⁴ cells/well) and cultured for 24 h at 37 °C with 5% CO₂ in a standard cell culture incubator. After 24 h of initial cell seeding, A549-AT cell cultures were pretreated with various concentrations of curcumin (15.62, 7.81, 3.90, 1.95, and 0.97 µg/mL), glycyrrhizin (4, 2, 1, 0.5, and 0.25 mg/mL), (-)-epigallocatechin (25, 12.5, 6.25, and 3.125 µg/mL), or harmaline (25, 12.5, 6.25, and 3.125 µg/mL). The compounds were diluted in Minimum Essential Medium (MEM) supplemented with 2% fetal bovine serum (FBS), 100 U/mL penicillin, and 0.1 mg/mL streptomycin (all from Thermo Fisher Scientific), and applied to the cells in a total volume of 500 µL. The cultures were incubated for two hours at 37 °C in a humidified atmosphere containing 5% CO₂. Following pretreatment, the inoculation medium was removed, and the cells were infected with 100 TCID₅₀ of SARS-CoV-2 (D614G, Omicron BA.5, or Omicron XBB.1) in the presence of various concentrations of curcumin (15.62, 7.81, 3.90, 1.95, and 0.97 µg/mL), glycyrrhizin (4, 2, 1, 0.5, and 0.25 mg/mL), (-)-epigallocatechin (25, 12.5, 6.25, and 3.125 µg/mL), or harmaline (25, 12.5, 6.25, and 3.125 µg/mL). Untreated cells were included as negative controls. After two days of incubation at 37 °C and 5% CO₂, the supernatants were collected and centrifuged to remove cellular debris. The clarified supernatants were then stored at −80 °C until further analysis. Viral titers were subsequently determined by endpoint dilution assay. In brief, serial dilutions of the cell culture supernatants (ranging from 1:100 to 1:10⁸) were added to confluent A549-AT cells seeded in 96-well microtiter plates (1.8 × 10⁴ cells per well). After three days of incubation at 37 °C in a humidified atmosphere with 5% CO₂, the inoculation medium was removed, and the cells were stained with 0.5% crystal violet (Carl Roth) dissolved in 20% methanol (Merck, Darmstadt, Germany). Following staining, cytopathic effects (CPEs) were assessed by light microscopy. All experiments were performed in triplicate. In Addition, we tested mock controls containing the respective vehicle at the highest final concentration used (DMSO or EtOH corresponding to the concentration at the highest compound dose).

### Quantification of SARS-CoV-2 RNA

The total RNA from the frozen cell culture supernatants was purified using the QIAamp Viral RNA Mini Kit (Qiagen, Hilden, Germany) according to the manufacturer’s instructions, including an initial DNase I treatment with the RNase-Free DNase Set (Qiagen) to remove any contaminating DNA. From the total RNA, 250 ng were reverse transcribed using the PrimeScript RT Master Mix (Takara, Kusatsu, Japan) for the relative quantification of SARS-CoV-2 M or N gene expression. For RT-qPCR, the GoTaq Probe qPCR Master Mix (Promega, Madison, WI, USA) was used according to the manufacturer’s protocol, with gene-specific primers and probes as previously described [35].

### Cytotoxicity assay

Possible cytotoxic effects of different concentrations of curcumin, glycyrrhizin, (–)-epigallocatechin, and harmaline on A549-AT cells were analyzed using the Orangu™ Cell Counting Solution (Cell Guidance Systems, Cambridge, UK), as previously described [27]. Therefore, different concentrations of curcumin (15.62 µg/mL, 7.81 µg/mL, 3.9 µg/mL, 1.95 µg/mL, 0.97 µg/mL), glycyrrhizin (4 mg/mL, 2 mg/mL, 1 mg/mL, 0.5 mg/mL, 0.25 mg/mL), (-)-epigallocatechin (25 µg/mL, 12.5 µg/mL, 6.25 µg/mL, 3.125 µg/mL), and harmaline (25 µg/mL, 12.5 µg/mL, 6.25 µg/mL, 3.125 µg/mL), all in MEM supplemented with 2% FBS, 100 U/mL penicillin, and 0.1 mg/mL streptomycin (each from Thermo Fisher Scientific), were added to confluent A549-AT cells grown on 96-well microtiter plates (2 × 10^4^ cells/well) and incubated at 37 °C and 5% CO_2_. At three different time points (24 h, 48 h, and 72 h), the medium was replaced with 10% Orangu™ cell counting solution and incubated for 60 min at 37 °C and 5% CO2. Following incubation, cell viability was measured using the Tristar 3 (Berthold Technologies, Oak Ridge, TN, USA) at an absorbance of 450 nm and normalized to untreated control cells. This experiment was performed in triplicate.

### Statistics

Statistical analysis was performed using GraphPad Prism version 10.2.3 (GraphPad Software, San Diego, CA, USA). For the evaluation of categorical variables, a Kruskal-Wallis test (a non-parametric one-way ANOVA) was applied. Two-sided p-values < 0.05 were considered significant.

## Results

### Dose-dependent neutralizing effects of glycyrrhizin, curcumin, harmaline, and (-)-epigallocatechin on SARS-CoV-2 in vitro

**Fig. 1:**
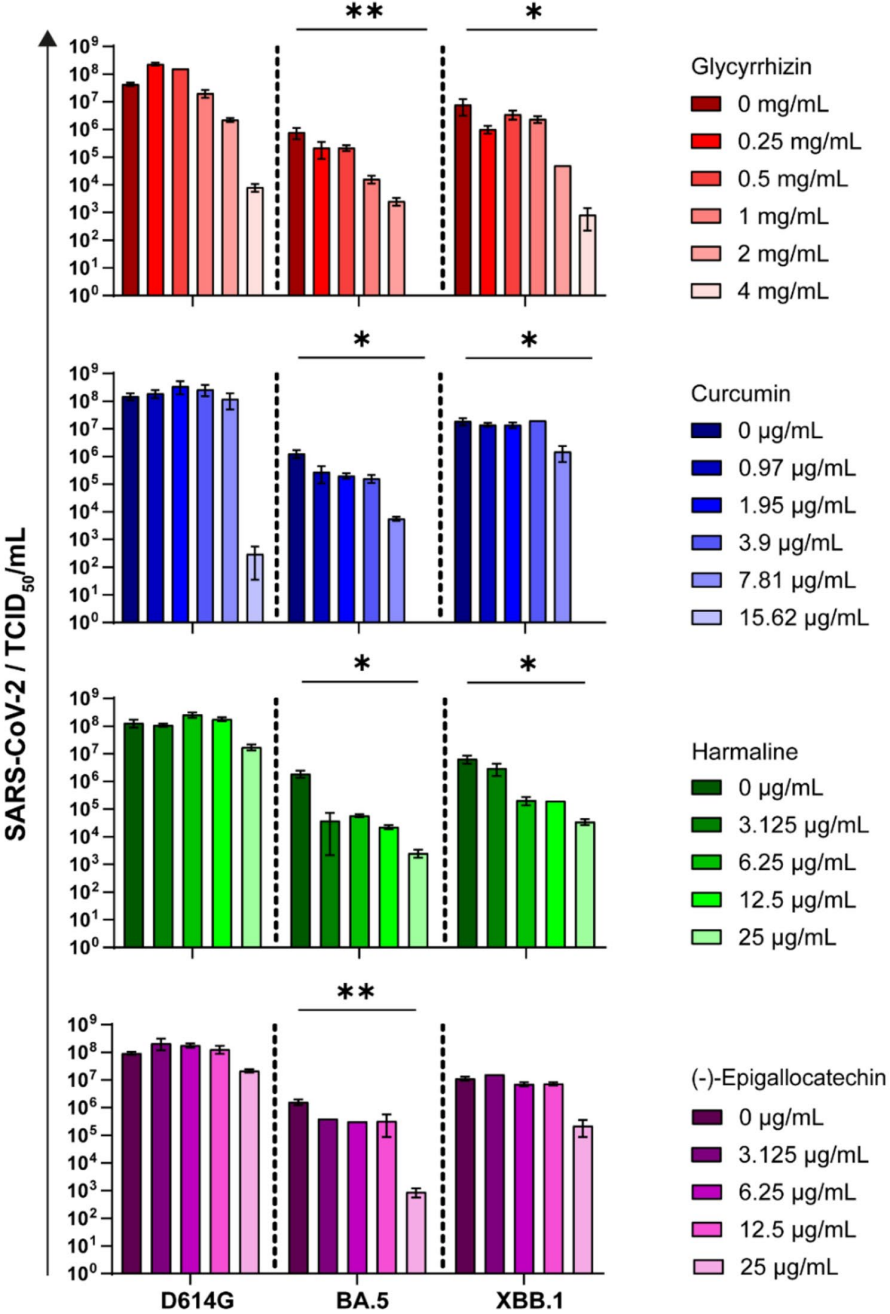
Glycyrrhizin, curcumin, harmaline and (-)-epigallocatechin reduced the viral load in a dose-dependent manner. A549-AT cells were pre-incubated with different concentrations of glycyrrhizin (0-4mg/mL), curcumin (0–15,62µg/mL), harmaline (0–25µg/mL) and (-)-epigallocatechin (0–25µg/mL) for two hours. Subsequently, the inoculation medium was removed, and the cells were infected with 100 TCID₅₀ of SARS-CoV-2 (D614G, Omicron BA.5, or Omicron XBB.1) in the presence of the indicated concentrations of curcumin, glycyrrhizin, harmaline or (-)-epigallocatechin. After 48 hours, the cell culture supernatants were harvested and the viral loads were determined by endpoint dilution. The experiments were performed in triplicate. Data are shown as mean and standard deviation (SD). Differences between each active ingredient and the medium control were analyzed using Kruskal-Wallis test (*p < 0.05; **p < 0.01)

To investigate the potential antiviral activities of glycyrrhizin, curcumin, harmaline, and (-)-epigallocatechin, various concentrations of each compound were tested against clinical isolates of SARS-CoV-2 (D614G, Omicron BA.5, and Omicron XBB.1) using a cell culture-based neutralization assay followed by an endpoint dilution assay. Glycyrrhizin reduced the replication of all tested SARS-CoV-2 variants in a dose-dependent manner. At a concentration of 4 mg/mL, glycyrrhizin decreased the viral load of the D614G variant by 4 log levels (*p* = 0.11). Furthermore, replication of the Omicron BA.5 variant was completely inhibited at 4 mg/mL glycyrrhizin (*p* = 0.0048). Replication of the Omicron XBB.1 variant was also significantly inhibited at this concentration (*p* = 0.018) (Fig. 1). Curcumin exhibited similar antiviral activity to glycyrrhizin, showing dose-dependent inhibition of SARS-CoV-2 replication. At 15.62 µg/mL, curcumin completely blocked replication of both the Omicron BA.5 (*p* = 0.0122) and XBB.1 (*p* = 0.0026) variants compared to untreated controls. While curcumin reduced the D614G variant’s viral load by nearly 6 log_10_ units at this concentration, the effect did not reach statistical significance (*p* = 0.60) (Fig. 1). Harmaline significantly reduced the viral replication of Omicron BA.5 at a concentration of 25 µg/mL (*p* = 0.0144) and Omicron XBB1 (*p* = 0.026) compared to the untreated control. In contrast, harmaline showed almost no effect against SARS-CoV-2 D614G, even at the highest concentration (Fig. 1). Of the compounds tested, (-)-epigallocatechin showed the weakest antiviral effect. Only a weak neutralizing effect of (-)-epigallocatechin was observed against D614G and Omicron XBB.1 at a concentration of 2 µg/mL. A significant reduction in viral load at a concentration of 25 mg/mL (-)-epigallocatechin was observed only against Omicron BA.5 (*p* = 0.0009) (Fig. 1). In vehicle-only controls containing DMSO or EtOH at the highest final concentrations used, no relevant effects on SARS-CoV-2 replication were observed.

### Dose-dependent inhibition of SARS-CoV-2 replication by glycyrrhizin, curcumin, harmaline, and (-)-epigallocatechin in vitro

To further validate the antiviral effects of glycyrrhizin, curcumin, harmaline, and (-)-epigallocatechin against SARS-CoV-2 variants D614G, Omicron BA.5, and Omicron XBB.1, SARS-CoV-2 RNA levels were quantified in cell culture supernatants from infected cell-cultures treated with each compound. Therefore, supernatants from A549-AT cells infected with 100 TCID_50_ of different SARS-CoV-2 isolates (D614G, Omicron BA.5, and Omicron XBB.1) and treated with various concentrations of glycyrrhizin (0–4 mg/mL), curcumin (0–15.62 µg/mL), harmaline (0–25 µg/mL), or (-)-epigallocatechin (0–25 µg/mL) were harvested after two days of incubation. Viral RNA was then isolated and reverse transcribed, and the total amounts of the SARS-CoV-2 M and N genes were quantified using RT-qPCR.

**Fig. 2:**
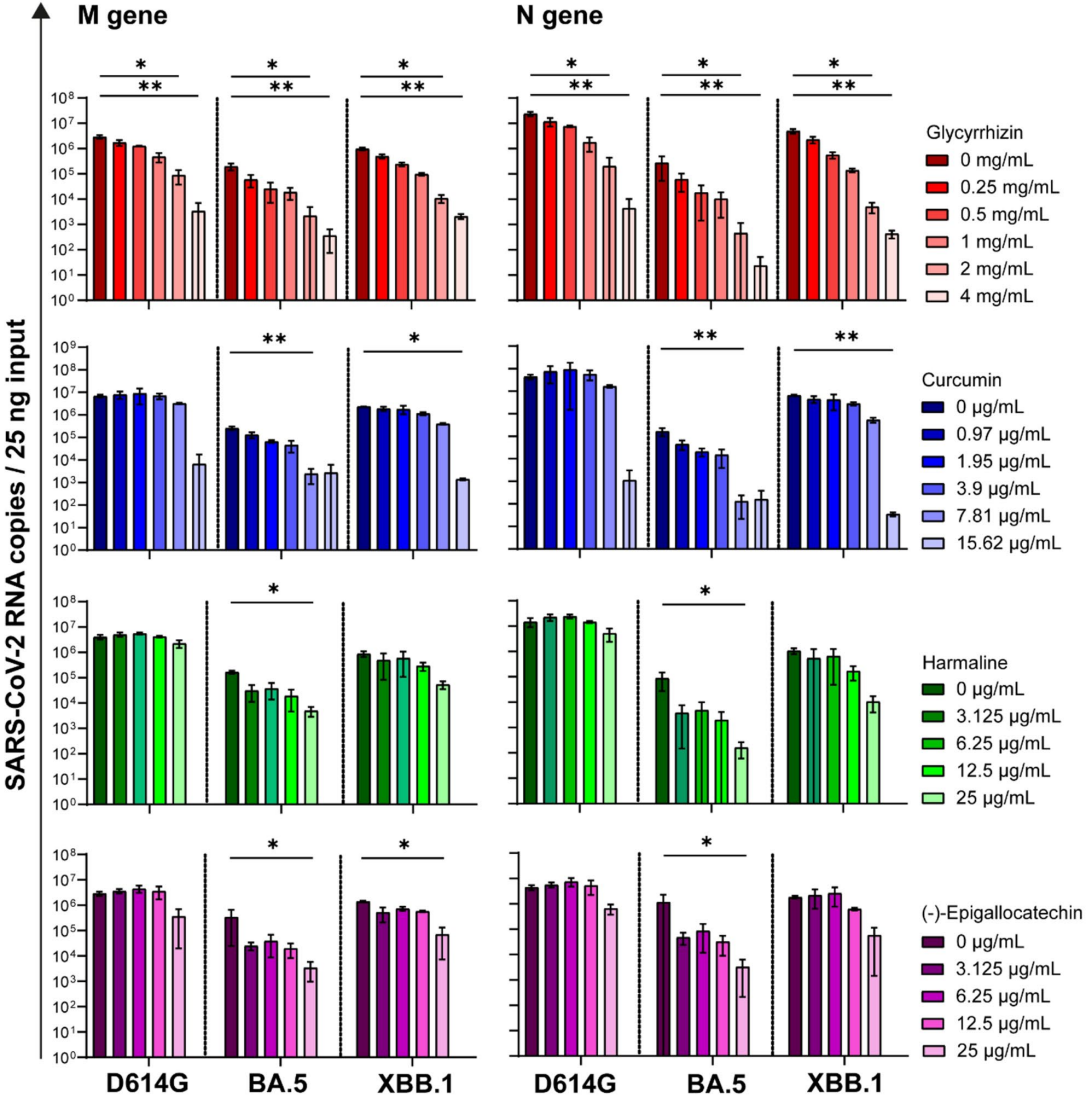
Glycyrrhizin, curcumin, harmaline and (-)-epigallocatechin reduced the SARS-CoV-2 RNA levels. A549-AT cells were pre-incubated with different concentrations of glycyrrhizin (0–4 mg/mL), curcumin (0–15.62 µg/mL), harmaline (0–25 µg/mL), and (-)-epigallocatechin (0–25 µg/mL) for two hours. Subsequently, the inoculation medium was removed, and the cells were infected with 100 TCID_50_ of SARS-CoV-2 (D614G, Omicron BA.5, or Omicron XBB.1) in the presence of the indicated concentrations of curcumin, glycyrrhizin, harmaline, or (-)-epigallocatechin. After 48 hours, the cell culture supernatants were harvested, and SARS-CoV-2 RNA was isolated and reverse transcribed. The number of M- and N-gene copies was determined using RT-qPCR. All experiments were performed in triplicate. Differences between each active ingredient and the medium control were analyzed using Kruskal-Wallis test (*p < 0.05; **p < 0.01)

Glycyrrhizin treatment reduced the copy number of SARS-CoV-2 M- and N- gene RNA in a dose-dependent and variant-independent manner. A significant reduction in SARS-CoV-2 M-gene RNA was observed for the variants SARS-CoV-2 D614G (4 mg/mL, *p* = 0.0034; 2 mg/mL, *p* = 0.038), Omicron BA.5 (4 mg/mL, *p* = 0.0066; 2 mg/mL, *p* = 0.037), and Omicron XBB.1 (4 mg/mL, *p* = 0.0029; 2 mg/mL, *p* = 0.03) (Fig. 2). Additionally, a significant decrease in N-gene RNA copy number was detected for SARS-CoV-2 D614G (4 mg/mL, *p* = 0.0034; 2 mg/mL, *p* = 0.038), Omicron BA.5 (4 mg/mL, *p* = 0.0066; 2 mg/mL, *p* = 0.037), and Omicron XBB.1 (4 mg/mL, *p* = 0.0029; 2 mg/mL, *p* = 0.03) (Fig. 2).

**Fig. 3:**
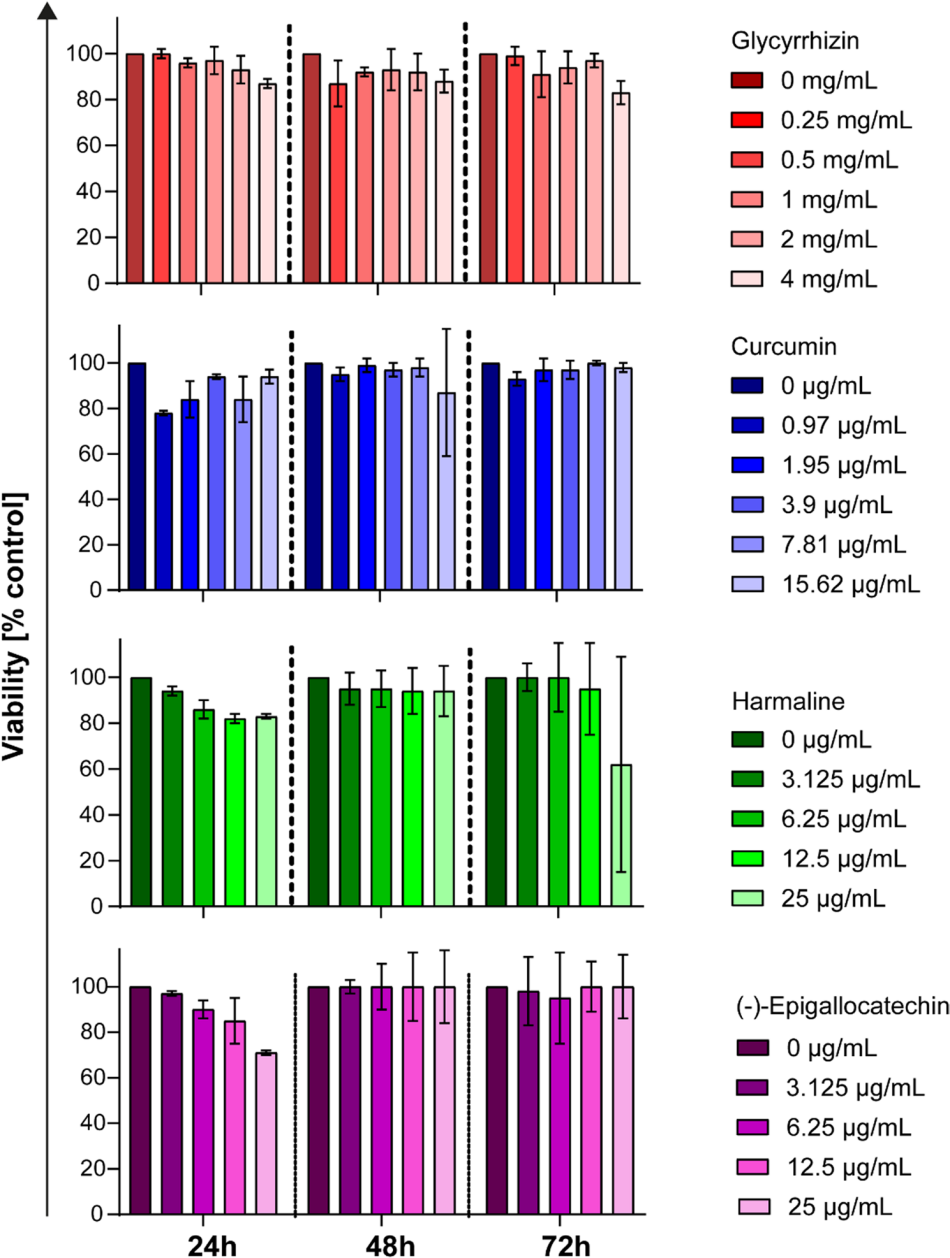
Glycyrrhizin, curcumin, harmaline, and (-)-epigallocatechin exhibited no toxicity toward A549-AT cells at the tested neutralizing concentrations. The potential cytotoxic effects of various concentrations of glycyrrhizin, curcumin, harmaline, and (-)-epigallocatechin on A549-AT cells were assessed using Orangu™ Cell Counting Solution (Cell Guidance Systems) after 24, 48, and 72 hours of incubation. All experiments were performed in triplicate. Differences between each active ingredient and the medium control were analyzed using Kruskal-Wallis test (*p < 0.05; **p < 0.01)

Curcumin demonstrated a dose-dependent reduction in M and N gene expression levels, comparable to the inhibitory effects observed with glycyrrhizin treatment. Curcumin treatment resulted in a marked reduction of SARS-CoV-2 D614G M gene RNA levels, though this decrease did not reach statistical significance (15.62 µg/mL; *p* = 0.089). In comparison, curcumin demonstrated significant reductions in M gene RNA levels for both Omicron BA.5 (7.81 µg/mL; *p* = 0.007) and Omicron XBB.1 (15.62 µg/mL; *p* = 0.014) (Fig. 2). Similar to its effect on M gene expression, curcumin treatment significantly reduced the number of N gene copies in both Omicron BA.5 (7.81 µg/mL; *p* = 0.0066) and Omicron XBB.1 (15.62 µg/mL; *p* = 0.0086). Furthermore, while higher concentrations of curcumin led to a multi-log reduction in the total N gene copy number of SARS-CoV-2 D614G, this decrease did not reach statistical significance (Fig. 3).

Although harmaline showed little to no effect on SARS-CoV-2 M and N gene copy numbers in the D614G variant, it reduced the total M gene copy number of both Omicron BA.5 and XBB.1 subvariants in a dose-dependent manner. Notably, a significant reduction in M gene RNA was observed only for Omicron BA.5 at 25 µg/mL (*p* = 0.0299). Accordingly, harmaline reduced the N gene copy number of both Omicron BA.5 and Omicron XBB.1 in a dose-dependent manner. At a concentration of 25 µg/mL, a significant reduction in the N gene copy number was observed for Omicron BA.5 (*p* = 0.0299) (Fig. 2).

Finally, (-)-epigallocatechin reduced the number of SARS-CoV-2 M and N gene copies in both Omicron BA.5 and XBB.1 in a dose-dependent manner. In contrast, only a weak effect was observed for the D614G variant. A significant reduction in the total amount of M gene RNA was found at an (-)-epigallocatechin concentration of 25 µg/mL for Omicron BA.5 (*p* = 0.003) and XBB.1 (*p* = 0.0233). The N gene copy number was significantly reduced only for Omicron BA.5 at a concentration of 25 µg/mL (*p* = 0.0233) (Fig. 2).

### Glycyrrhizin, curcumin, harmaline, and (-)-epigallocatechin exhibited no cytotoxicity

The cytotoxic potential of glycyrrhizin, curcumin, harmaline, and (-)-epigallocatechin on A549-AT cells was evaluated by assessing cell viability. Confluent A549-AT cells were treated with varying concentrations of glycyrrhizin (0–4 mg/mL), curcumin (0–15.62 µg/mL), harmaline (0–25 µg/mL), and (-)-epigallocatechin (0–25 µg/mL). Cell viability was measured after 24, 48, and 72 h. After 48 h, the incubation period used in neutralization assays to assess antiviral effects, no cytotoxic effects were observed for any of the compounds. Even after 72 h of incubation with the highest concentrations of each compound, no toxic effects were detected. In conclusion, glycyrrhizin, curcumin, harmaline, and (-)-epigallocatechin were able to neutralize SARS-CoV-2 D614G and its Omicron variants BA.5 and XBB.1 at subtoxic concentrations (Fig. 3).

## Discussion

SARS-CoV-2 remains a global health challenge, causing severe courses of COVID-19. Despite widespread vaccination, breakthrough infections continue to occur due to ongoing viral mutation, highlighting the urgent need for effective antiviral treatments against emerging variants. Natural compounds derived from medicinal herbs may offer a broadly effective, variant-independent treatment option, particularly in regions with limited access to new therapies. Previous studies have shown that agents from these sources can inhibit replication of early SARS-CoV-2 variants. However, their effectiveness against newly emerging variants has not been fully explored. In this study, we analyzed the antiviral efficacy of glycyrrhizin, curcumin, harmaline, and (-)-epigallocatechin against SARS-CoV-2 variants D614G, Omicron BA.5, and Omicron XBB.1.

The strongest antiviral effects were observed with glycyrrhizin and curcumin. At concentrations of 4 mg/mL for glycyrrhizin and 15.6 µg/mL for curcumin, replication of the SARS-CoV-2 variants D614G, Omicron BA.5, and Omicron XBB.1 was effectively inhibited. Harmaline exhibited antiviral activity against the Omicron BA.5 and Omicron XBB.1 variants but showed no effect against the D614G variant. In contrast, (-)-epigallocatechin showed minimal antiviral activity and only slightly inhibited the replication of SARS-CoV-2 Omicron BA.5. The results of our study are consistent with and extend the findings of previous research, in which the antiviral activities of these compounds have been described.

Glycyrrhizin, a triterpenoid saponin derived from licorice root (Glycyrrhiza glabra), has previously been reported to have broad-spectrum antiviral activity. Early in the COVID-19 pandemic, glycyrrhizin was identified as a potential inhibitor of SARS-CoV-2 [27] based on its prior efficacy against SARS-CoV-1 [19]. Clinical data suggest that glycyrrhizin, often administered in combination with other bioactive compounds, can modulate inflammatory responses in vivo, including reductions in interleukin levels and, in some studies, shortened recovery times in COVID-19 patients [36, 37]. Adverse effects such as pseudo-hyperaldosteronism, hypertension, or hypokalemia generally occur only after prolonged or high-dose use and are well described in the literature, making them predictable and clinically manageable [38]. Overall, glycyrrhizin is considered a well-tolerated compound when used at appropriate therapeutic doses [39]. We previously demonstrated that glycyrrhizin inhibits SARS-CoV-2 replication by targeting the viral main protease (Mpro) [27], which is essential for the proteolytic processing of the viral polyproteins pp1a and pp1ab into the non-structural proteins required for viral replication [40–42]. Because this mechanism does not involve the spike protein, it is largely independent of spike-associated mutations, providing a plausible explanation for the broad antiviral activity of glycyrrhizin across different SARS-CoV-2 variants. Similarly, curcumin has been reported to exhibit antiviral activity against a wide range of viruses and, in our earlier work, was shown to neutralize SARS-CoV-2 in vitro [28]. Available in silico and in vitro data suggest a multifactorial mode of action, including interactions with the viral main protease and the spike protein, which may support a broadly conserved antiviral effect while still allowing for variant-dependent modulation [43]. In the present study, antiviral effects against D614G were less pronounced in A549-AT cells than previously observed in Vero E6 cells. This may be explained by the overexpression of ACE2 and TMPRSS2, leading to enhanced viral entry and higher viral loads that reduce measurable neutralization efficiency. In contrast, the Omicron variants tested showed strong susceptibility to glycyrrhizin and curcumin in this model. For (−)-epigallocatechin and harmaline, mechanistic data remain scarce, and further fundamental studies will be required to clarify their molecular targets and potential variant-specific effects. Previous in silico and in vitro studies have suggested that curcumin may interact with key viral proteins, including the SARS-CoV-2 main protease and the spike protein [44, 45]. This multifactorial mode of action may account for the variant-independent antiviral efficacy observed with curcumin in this study. Despite its therapeutic potential, the clinical use of curcumin is limited by its poor systemic bioavailability, which may reduce its effectiveness in vivo. Co-administration with piperine, a major constituent of black pepper, has been shown to markedly enhance absorption and systemic availability in humans [46]. Several recent clinical studies reported that curcumin combined with piperine may reduce selected clinical symptoms, markers of morbidity, or inflammatory parameters in COVID-19 patients, although the evidence is still heterogeneous and larger, well-controlled trials are required to confirm these findings [47]. Harmaline, a β-carboline alkaloid found in *Peganum harmala*, exhibited weaker antiviral effects compared to glycyrrhizin and curcumin. It showed activity against the SARS-CoV-2 Omicron variants BA.5 and XBB.1, but not against the D614G variant. *Peganum harmala* and its active ingredients have a long history in the treatment of various human diseases, including cough, diabetes and asthma [48]. Harmaline, one of the main alkaloids from *Peganum harmala*, is known for its antifungal, antiparasitic, and antimicrobial activities. Its antiviral effects have also been described against influenza and herpes simplex viruses [18, 49]. However, its direct antiviral potential, especially against coronaviruses, remains poorly characterized. Previous studies have demonstrated that several bioactive alkaloids derived from *Peganum harmala*, including harmaline, can interact with different SARS-CoV-2 proteins, although with variable and generally moderate binding affinities. The modest antiviral activity of harmaline observed in our study may therefore reflect relatively weak or nonspecific interactions with viral or host targets. Notably, antiviral effects were detectable only at higher concentrations and were restricted to the Omicron BA.5 and XBB.1 variants, while no activity was observed against the D614G variant [50, 51]. This variant-specific activity may be explained by mutations present in Omicron variants that alter viral protein conformation, surface accessibility, or host–virus interactions, thereby enhancing the interaction of harmaline with relevant targets. Nevertheless, the exact molecular basis of this effect remains unclear. Additional mechanistic and structural studies will be required to identify the primary targets of harmaline, to determine how Omicron-associated mutations influence these interactions, and to assess potential synergistic effects with other antiviral compounds.

(-)-Epigallocatechin, a polyphenol from green tea, was the least effective in our study. Although related catechins, such as epigallocatechin gallate (EGCG), have been reported to inhibit SARS-CoV-2 in vitro, the lack of galloyl moiety in EGC may significantly reduce its binding affinity to viral targets [52]. This observation aligns with recent studies indicating that, while EGCG (epigallocatechin gallate) and some derivatives can strongly suppress infectivity of certain SARS-CoV-2 variants, (-)-epigallocatechin itself does not significantly affect the infectivity of Omicron subvariants [53]. The limited antiviral effect observed for (-)-epigallocatechin, together with the currently unclear underlying molecular mechanism, underscores the need for further mechanistic studies to better understand its potential activity and target interactions. Such investigations will be essential to determine whether structural modifications or derivatives of (-)-epigallocatechin could yield stronger antiviral effects and to clarify whether its activity may depend on specific SARS-CoV-2 variants.

## Conclusions

Taken together, our results confirm the potential of glycyrrhizin and curcumin as promising natural antivirals against SARS-CoV-2, with harmaline showing more limited but still noteworthy activity. These compounds interact with various viral proteins of SARS-CoV-2, including the Omicron variants BA.5 and XBB.1, and exert additional immunomodulatory effects such as anti-inflammatory and antioxidant activities. Their variant-independent efficacy—likely due to multifactorial mechanisms of action—underscores the importance of structural features in determining antiviral potency. In particular, in low- and middle-income countries, where access to newly developed antiviral drugs may be limited, these natural agents could represent affordable and accessible options for the supportive treatment of COVID-19. Combination treatment with glycyrrhizin and curcumin may enhance antiviral efficacy through complementary mechanisms. Glycyrrhizin primarily targets the viral main protease, while curcumin exhibits multifactorial activity, including effects on Mpro and viral entry. Targeting distinct stages of the viral life cycle may increase antiviral potency and allow lower concentrations of each compound, potentially reducing dose-related side effects. Nevertheless, further controlled combination and mechanistic studies are required to confirm synergistic effects and evaluate clinical relevance. Beyond mechanistic studies, pharmacokinetic and clinical investigations will be required to determine the translational relevance and in vivo effectiveness of these compounds, as in vitro concentrations cannot directly predict achievable or effective levels in humans.

## Data Availability

The datasets used and/or analyzed during the current study are available from the corresponding author on reasonable request.

## Abbreviations

ACE 2: Angiotensin Converting Enzyme 2
ANOVA: Analysis of Variance
ARDS: Acute Respiratory Distress Syndrome
CPE: Cytopathic Effect
COVID19: Coronavirus disease 2019
DMSO: Dimethyl sulfoxide
EGC: (-)-epigallocatechin
EGCG: Epigallocatechin gallate
FBS: Fetal bovine serum
HIV: Human Immunodeficiency Virus
HSV-1: Herpes Simplex Virus type 1
HSV-2: Herpes Simplex Virus type 2
MEM: Minimum Essential Medium
Mpro: Main proteases
Nsp: Non-structural proteins
pp: Polyprotein
RNA: Ribonucleic acid
RSV: Human respiratory syncytial virus
SARS-CoV-1: Severe acute respiratory syndrome coronavirus 1
SARS-CoV-2: Severe acute respiratory syndrome coronavirus 2
TCID_50_: Tissue Culture Infectious Dose 50
TMPRSS2: Transmembrane protease serine subtype 2
WHO: World Health Organization
